# Density and temperature controlled fluid extraction in a bacterial biofilm is determined by poly-*γ*-glutamic acid production

**DOI:** 10.1038/s41522-022-00361-5

**Published:** 2022-12-17

**Authors:** Ryan J. Morris, David Stevenson, Tetyana Sukhodub, Nicola R. Stanley-Wall, Cait E. MacPhee

**Affiliations:** 1 National Biofilms Innovation Centre, School of Physics and Astronomy, The University of Edinburgh, Edinburgh, EH9 3FD UK; 2 Division of Molecular Microbiology, School of Life Sciences, University of Dundee, Dundee, DD1 5EH UK

**Keywords:** Biofilms, Microbial communities

## Abstract

A hallmark of microbial biofilms is the self-production of an extracellular molecular matrix that encases the resident cells. The matrix provides protection from the environment, while spatial heterogeneity of gene expression influences the structural morphology and colony spreading dynamics. *Bacillus subtilis* is a model bacterial system used to uncover the regulatory pathways and key building blocks required for biofilm growth and development. In this work, we report on the emergence of a highly active population of bacteria during the early stages of biofilm formation, facilitated by the extraction of fluid from the underlying agar substrate. We trace the origin of this fluid extraction to the production of poly-*γ*-glutamic acid (PGA). The flagella-dependent activity develops behind a moving front of fluid that propagates from the boundary of the biofilm towards the interior. The extent of fluid proliferation is controlled by the presence of extracellular polysaccharides (EPS). We also find that PGA production is positively correlated with higher temperatures, resulting in high-temperature mature biofilm morphologies that are distinct from the rugose colony biofilm architecture typically associated with *B. subtilis*. Although previous reports have suggested that PGA production does not play a major role in biofilm morphology in the undomesticated isolate NCIB 3610, our results suggest that this strain produces distinct biofilm matrices in response to environmental conditions.

## Introduction

A common strategy employed by bacteria to mitigate stresses imposed by their environment is to co-exist in sessile communities known as biofilms. The transition from unicellular to multi-cellular life allows the residents to coordinate responses to stimuli, share metabolic burdens^1^, and protect against external attack by predators^2,3^ or antimicrobial agents^4,5^. This behaviour is ubiquitous across the microbial world and a clear understanding of biofilm genesis, development, and maturation is important both from a fundamental microbiological perspective, but also due to their impact on many industrial, clinical, and biotechnological sectors. For example, biofilms act as sources for many chronic infections and their physical characteristics make them difficult to eradicate^6,7^. This intransigence can also impact industrial processes, where biofilms may result in pipe blockages, induce corrosion, or contaminate products^8–10^. However, while there are many negative consequences of biofilm formation, microbial biofilms play vital roles in waste water treatment and other bioremediation processes^11–14^ and understanding their formation as well as their disruption is of fundamental interest.

*Bacillus subtilis* is a Gram-positive bacterium that has been extensively used as a model organism to investigate the genetic regulation and molecular mechanisms of biofilm formation^15,16^. The major components of the matrix produced by *B. subtilis* are the fibrillar protein TasA^17^, the hydrophobin-like protein surfactant BslA^18–20^, and the polysaccharide synthesized by products of the *epsA-O* operon^21^. One of the principle regulatory pathways that controls the expression of these components is modulated by the transcription factor Spo0A, with moderate levels of phosphorylated Spo0A activating transcription of the *sinI-sinR* operon^22,23^. SinR is a DNA-binding transcription factor that controls matrix production by interacting with the *epsA-O* and the *tapA-sipW-tasA* promoters^24^. When SinR binds to its antagonist proteins (SinI and SlrR), repression is alleviated from these operons and biofilm formation can proceed^25,26^.

In this work we report on the observation of multiple travelling fluid fronts that develop during the early stage of *B. subtilis* colony biofilm formation. Following initial deposition of founding cells, a highly motile population of bacteria emerges that swims in fluid extracted from the underlying agar substrate. We genetically link this moving front of fluid to the production of the polymer poly-*γ*-glutamic acid (PGA). We find the influx of fluid is dependent on both bacterial density and environmental temperature, and that the extent of fluid infiltration is in turn modulated by the production of extracellular polysaccharide (EPS). The production of PGA at higher temperatures, and the concomitant extraction of fluid, significantly impacts the mature biofilm morphology, which diverges from the typical structure associated with *B. subtilis* NCIB 3610. Our results suggest that *B. subtilis* has the ability to produce an alternative extracellular matrix in response to the environmental conditions.

## Results

### Deposition and imaging of growing biofilms

**Fig. 1:**
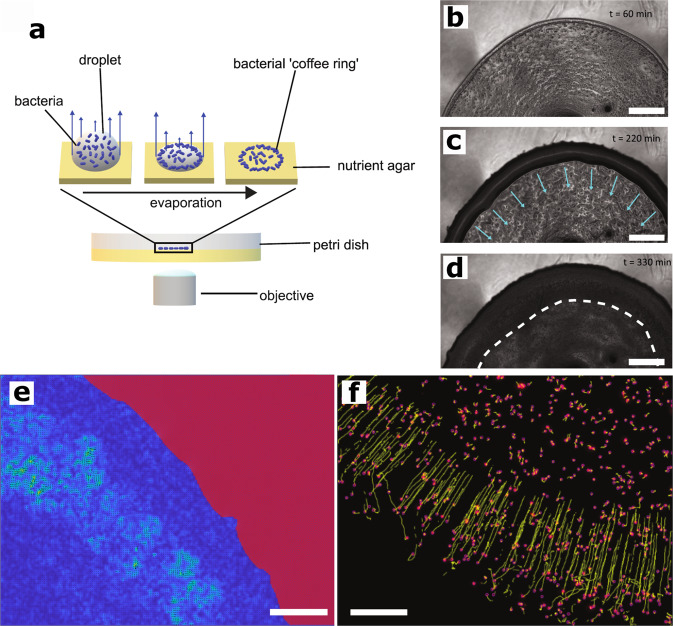
Schematic of the experiment and emergence of a travelling front of motile cells. **a** A 3 μL droplet of a bacterial suspension is deposited onto the surface of MSgg agar and allowed to dry. Due to the differential rates of evaporation across the surface of the droplet (blue arrows), capillary flows are induced in the interior of the droplet. This allows for the transport of bacteria from the interior of the droplet to the edge where they become deposited. The effect is an accumulation of a higher-density region of bacteria at the contact line between the droplet and solid agar. **b** This higher-density region of bacteria can be seen at the edge of the colony (*t* = 60 min). **c** After three hours of incubation and growth a darker region develops centered upon the edge of the emerging biofilm. We find that this zone is fluid and begins to move inwards towards the center of the colony (blue arrows; *t* = 220 min). In this zone we observe active and motile cells. **d** After another two hours, the front stops moving inwards, indicated by the white dashed line, and the motility stops (*t* = 330 min). Scale bars (**b**)–(**d**) are 500 μm. **e** PIV analysis showing the velocity magnitude field of a wild-type biofilm over an interval of two frames (10 fps). The blue region is the colony biofilm; the red region is the agar. Image captured ~4.5 h after deposition. **f** Using particle tracking software, the beads are located (pink) and their motion tracked over time (yellow lines).The majority of beads move linearly at a constant speed towards the interior of the colony. Image captured ~200 min after deposition. Scale bars (**e**)–(**f**) 100 μm.

At the beginning of each experiment, a 3 μL suspension of *B. subtilis* cells (OD_600_ = 1) was deposited onto MSgg agar, a biofilm-promoting minimal media^27^. After inoculation the droplet evaporates, which results in a ‘coffee ring’ deposition pattern: a high-density ring of bacterial cells forms at the edge of the initial droplet while the interior is more sparsely populated (Fig. 1a). This distribution of the founding cells is caused by the differential rate of evaporation across the droplet, driving capillary flows that transport cells from the interior to the boundary between the droplet and solid agar^28^. Our initial experiments were performed using the wild-type isolate NCIB 3610 grown at 38 ^∘^C while time-resolved images were collected. The images were captured by imaging through the agar substrate, thus we observed the dynamics of growth on the underside of the biofilm (Fig. 1a). We acquired images for the initial ~6 h of growth (Fig. 1b–d). The high-density ‘coffee ring’ region is clearly identifiable by the large accumulation of bacterial cells near the outer boundary of the colony (Fig. 1b). This high-density region of bacteria appears to be multi-layered with a width of 75–100 μm.

### Emergence of a fluid flux that induces motility within the early biofilm

After NCIB 3610 has grown for ~3 h we observe a zone, emerging from the high-density region, that is visually different to the interior of the colony (c.f. the dark annulus at the edge compared to the lighter interior in Fig. 1c). This darker zone grows and moves inwards as a travelling front towards the colony centre (Supplementary Movie 1). Within this zone we observe coherent patterns of self-organised motion such as swirls and vortices, reminiscent of structures observed in active turbulent systems (Supplementary Movie 2)^29,30^ which indicate that the bacteria are now in a fluid environment. We used Particle Image Velocimetry (PIV) to further assess this motion. We found localized regions of high vorticity, and velocities ranging from 1 to 10 μm/s with a mean of 2.1 μm/s (Fig. 1e, Supplementary Fig. 1, Supplementary Movies 3, 4). To determine whether these emergent patterns were due to passive advection of the bacteria due to fluid flows or whether bacterial motility was involved, we performed similar experiments and PIV on a non-motile strain that possesses a deletion of *motB*, a flagellar stator element required for flagellar rotation. We found that we do not observe vortices as we did with NCIB 3610 (Supplementary Fig. 2). Moreover, the average velocity for the *motB* strain is an order of magnitude less than NCIB 3610 (Supplementary Figs. 2, 3). These observations support the idea that active flagella or motility are important for the formation of these dynamical features. Whether these patterns are an example of active turbulence due to bacterial motility^29^, or due to a coupling between flagellar beating and the flow of fluid into the colony from the agar below is beyond the scope of this work. Regardless, these results show that the bacteria at this phase of colony growth are in a fluid environment. This cessation of motility/activity also occurs as a propagating front (the “motility arrest front"), however it travels much faster than the initial propagation of fluid, and predominantly moves from the interior ring of the annulus back towards the outer initial coffee ring (for annotated movies of these dynamics see Supplementary Movies 5 and 6).

To further examine this fluid front, we added 2 μm fluorescent beads to the suspension of bacteria that were deposited at the beginning of the experiment (Fig. 1e, Supplementary Movie 7). With the influx of fluid, the beads are pushed along at a constant speed of ≈2.5 μm min^−1^ (Fig. 1f). Thus, the motion of this front has the ability to displace and mechanically push beads, and likely cells, towards the center of the colony. At later stages some beads become erratic in their movement, likely indicating beads that are set in motion by the swimming action of the bacteria in a fluid environment; a further portion of the beads remains embedded within the bacterial mass.

### Extracellular polysaccharide production mitigates the fluid flux

**Fig. 2:**
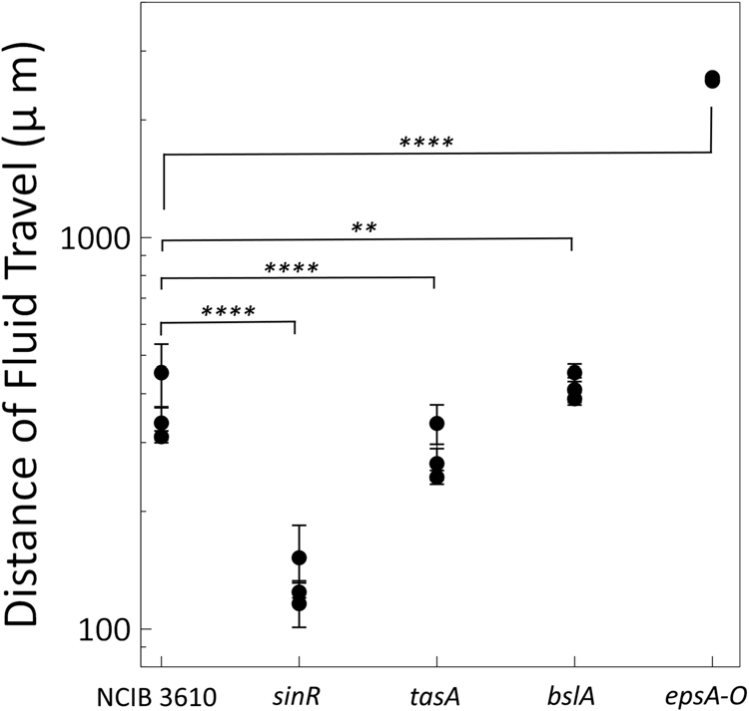
Matrix production can modify the spatial extent of fluid invasion. Plotted is the average distance of fluid travel as measured from the edge of the biofilm. Note the y-axis is on a log-scale to show the differences between the NCIB 3610, *sinR*, *tasA*, and *bslA* and *epsA-O* strains. Each data point is the mean fluid travel distance averaged over 10 spatial points across an individual biofilm (*N* = 3 for each strain). Error bars represent standard deviation. It should be noted for the *epsA-O* strain, the distance travelled by the fluid simply reflects the size of the colony since the fluid emanating from the boundary is not confined to an annulus but meets in the center. Here we report distances for three separate experiments. An unpaired *t*-test between NCIB 3610 and each matrix mutant gives *p*-values of ****p < 0.0001 (*sinR*), ****p < 0.0001 (*tasA*), **p = 0.0025 (*bslA*, ****p < 0.0001 (*epsA-O)*). *p* < 0.05 is considered statistically significant.

We consistently observed the formation of a fluid annulus beginning at the outer edge of the biofilm, rather than the swamping of the whole colony with fluid. We hypothesise that the restriction of the fluid to an outer annulus is due to extracellular matrix production within the central region of the biofilm, preventing further incursion of fluid. We performed the same experiment described in Fig. 1a using a strain in which the key repressor of biofilm formation (*sinR*), is deleted. Without *sinR*, the bacteria over-produce the extracellular matrix and highly wrinkled colony biofilms occupy a smaller footprint (Supplementary Fig. 4)^24^. As predicted, the maximum distance that the fluid encroached into the interior of the biofilm relative to the outer edge of the colony (Supplementary Figs. 5, 6; Supplementary Movie 8) in the *sinR* strain was approximately 3 times less than NCIB 3610 strain (Fig. 2).

Next, we wished to determine if one or more individual components of the matrix are dominant in controlling the extent of the fluid flux. We performed analogous experiments on strains with deletions in genes responsible for the production of BslA (*bslA*), TasA (*tasA*), and EPS (*epsA-O*), again measuring the distance of fluid travel. We found that the fluid traveled further in the *bslA* strain compared to NCIB 3610, while the fluid flow in the *tasA* strain traveled less (Fig. 2, Supplementary Figs. 5, 7, 8; Supplementary Movies 9, 10, 11). While differences between NCIB 3610 and these matrix components are statistically significant, the fluid is still contained to an annular region within the colony. For the *epsA-O* strain however, the fluid was not confined to an annular region but instead propagated across the entire radius of the colony (Fig. 2, Supplementary Movie 12) and with observable cell activity everywhere. The activity was highly dynamic and we observed vortex formation characteristic of active turbulence (Supplementary Movie 13). Again, we performed PIV analysis and found regions of high vorticity and a velocity distribution similar to the wild-type strain (Supplementary Fig. 9, Supplementary Movies 14, 15). The motility arrest front is more apparent in the *epsA-O* mutant and, like the fluid propagation front, travels across the entirety of the colony (for edited and annotated movies of the *epsA-O* biofilm dynamics see Supplementary Movies 16 and 17). Taken together our data demonstrate that EPS is the primary extracellular matrix component that controls the extent of fluid invasion into the biofilm.

### Whole biofilm imaging of epsA-O strain

**Fig. 3:**
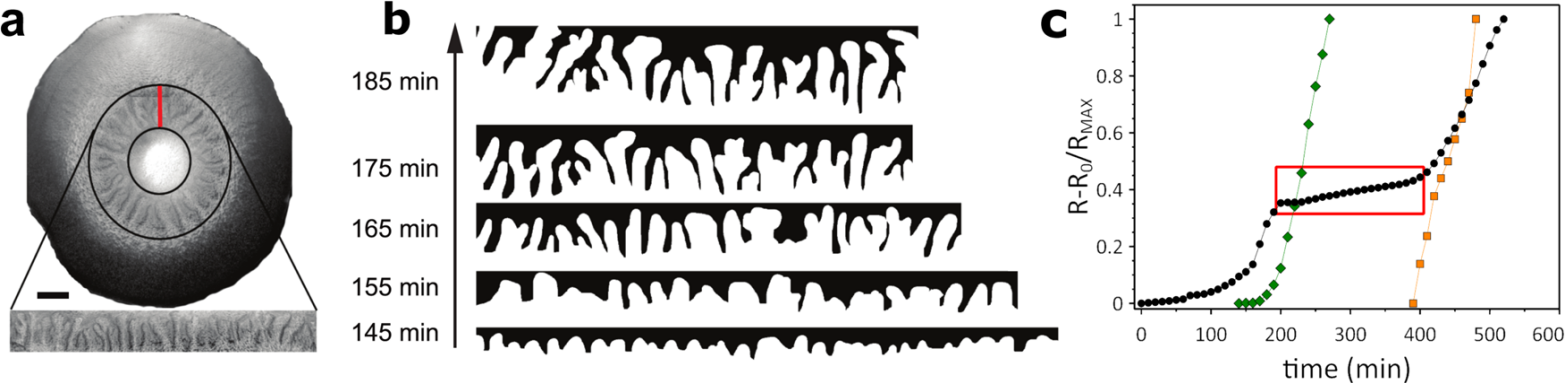
Large scale dynamics in polysaccharide-minus biofilms. **a** An example of a microscopy image of an entire *epsA-O* biofilm (scale bar is 500 μm). Annular black region defines the area where we observe finger-like instabilities. This annular region is made linear for ease of visualization; a cut is made at the red line and the circular region is `unrolled' to form a linear region. **b** Binarized images of the fingers over time for the biofilm in (**a**). **c** The normalized displacement as a function of time for the outer edge of the colony (black circles), fluid front (green diamonds) and motility arrest front (orange squares). A plateau in the edge expansion occurs coincidentally with the onset of fluid propagation (which we define as the `fluidisation plateau'; red box). The growth recommences after motility becomes arrested. The green curve corresponds to the time when we observe the fingers develop in (**a**).

The *epsA-O* strain allowed us to track the flux of fluid across the entire colony in a time-resolved manner and learn more about the fluid extraction process. We noticed a finger-like instability develops as the fluid propagates inwards (Fig. 3a; Supplementary Movies 8, 16–18). As the fluid pushes in towards the center of the colony the fluid front becomes unstable, forming increasingly large fingers over time (Fig. 3a, b). The patterns formed by these instabilities is reminiscent of the wrinkling pattern observed in mature biofilms.

Imaging the fluid flow in colony biofilms in this manner allowed us to fully track three key features of the dynamics: (1) the distance travelled by the expanding outer edge of the growing colony; (2) the distance travelled by the fluid flux, as above; and (3) the motility arrest front. All measurements were taken relative to the initial position (*r*_0_) of each feature, and the relative distances were normalized by their maximum value to allow direct comparison of their time-dependent evolution. Firstly, after a lag time, the outer edge of the colony begins to expand at a constant rate, as shown in Fig. 3c. However, this outward expansion slows down and then stalls at precisely the time that we see the fluid front begin to propagate from the coffee ring into the interior of the colony. We suggest that the influx of liquid causes a “fluidisation” of the interior biofilm mass, and the resulting lack of mechanical robustness prevents the further outward expansion of the biofilm. It is only with the onset of the motility arrest front—and re-solidification of the interior—that further expansion at the outer edge of the biofilm can occur. We refer to this pause in biofilm expansion due to the influx of fluid as the ‘fluidisation plateau’ (red box, Fig. 3c), and we can use it to probe changes in biofilm properties.

It is striking that the fluid front predominantly moves radially from the ‘coffee ring’ into the centre of the colony. This suggests that the molecular species responsible for fluid extraction is produced in a cell density-dependent manner. To test this hypothesis we prepared colony biofilms with and without an initial high-density region, by spin-coating a suspension of bacteria onto the agar. Samples with a heterogeneous distribution of cells (coffee-ring-like) always extract fluid with a directionality: fluid always propagates from high density to low density regions (Supplementary Movies 19, 20). In contrast, a homogeneous deposit of cells eventually results in fluid that enters into the colony everywhere simultaneously, with no direction of propagation (Supplementary Movies 21, 22). These findings indicate that there exists some critical cell density required to induce fluid extraction.

### Polyglutamic acid is the agent that induces the fluid flux into the biofilm

**Fig. 4:**
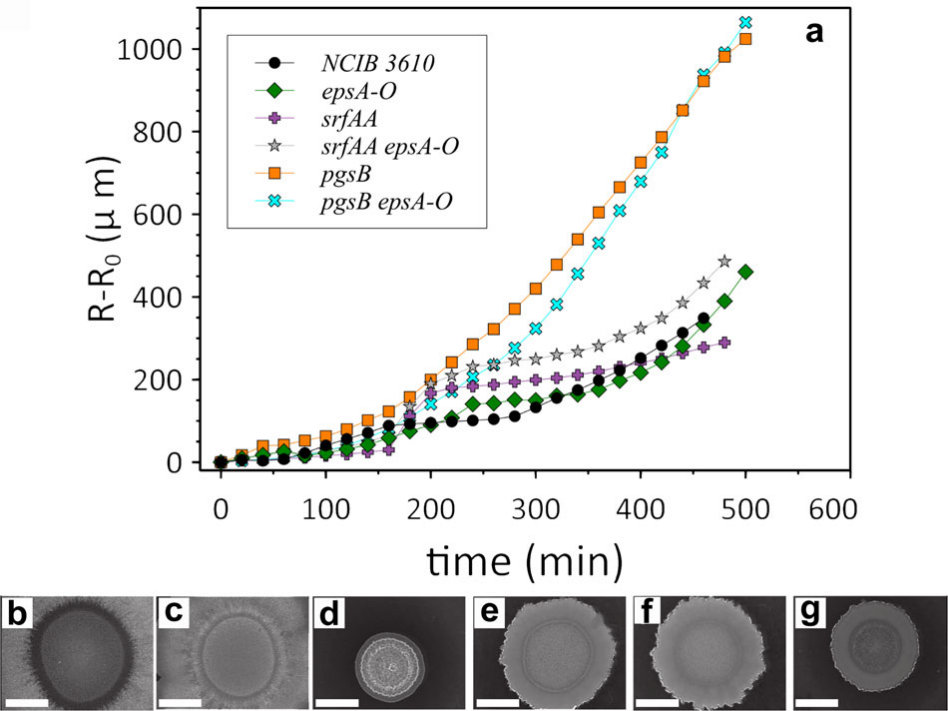
Fluid extraction is driven by PGA production. **a** Measurements of the outer edge displacement at 38 ^∘^C show that NCIB 3610 (black circles), *epsA-O* (green diamonds), the *srfAA* (purple plus), and *srfAA epsA-O* (grey star) strains possesses the characteristic `fluidization plateau'. The *pgsB* minus (orange square), and *pgsB epsA-O* minus strain (cyan cross) that cannot produce PGA did not exhibit this behaviour. Colony morphology after 48 hours incubation at 38 ^∘^C of **b** NCIB 3610, **c** *pgsB,* **d** *srfAA,* **e** *epsA-O*, **f** *pgsB epsA-O*, and **g** *srfAA epsA-O*. Scale bar is 5 mm.

We wished to identify the molecular species responsible for driving the fluid flux and subsequent activity and growth dynamics. Surfactin was an obvious candidate: it is a lipopeptide produced by *B. subtilis* that is a powerful biosurfactant^31^ and a potent anti-microbial agent^32^. Surfactin production in *B. subtilis* biofilms facilitates colony spreading^33^ and is important in the osmotic extraction of fluid from the underlying agar substrate^34^. To test whether surfactin is the causative agent of the dynamics we observe we deleted *srfAA* from the wild-type and *epsA-O* background strains. As a diagnostic we measured the relative displacement, *r*, of the biofilm edge relative to the initial position of the edge, *r*_0_ as a function of time (Fig. 4a). If surfactin is responsible for the fluid flux into the growing biofilm, deletion of *srfAA* should result in unimpeded colony expansion. However, we found that both *srfAA* and *srfAA epsA-O* strains exhibited the characteristic ‘fluidisation plateau’ displayed by the parental strain (Fig. 4a); hence surfactin is not the causative agent of the observed fluid extraction.

Poly-*γ*-glutamic acid is a naturally occurring biopolymer consisting of repeating units of L-glutamic acid, D-glutamic acid or both, produced by species of *Bacillus*^35,36^. PGA production is density-dependent^37^, consistent with our observations, and has humectant properties^38,39^. To test whether PGA is responsible for the fluid extraction, we deleted *pgsB* from the wild-type and *epsA-O* background strains. PgsB is the essential synthetase required for PGA production^40^. We observed a fluidisation plateau in the colony expansion for the wild-type and *epsA-O* strains, but not for the two *pgsB* mutants (Fig. 4a). When PGA is absent, the biofilm does not ‘fluidise’ and biofilm expansion continues uninterrupted. We therefore conclude that PGA is the molecular agent that extracts fluid from the substrate, driving the onset of motility and biofilm fluidisation.

We also imaged the biofilm morphology after 48 hours of incubation and found that the wild-type and *pgsB* mutant are morphologically similar (Fig. 4b, c). The two *epsA-O* strains are likewise comparable, but different to the wild-type morphology (Fig. 4e, f). For completeness, we also imaged the surfactin mutant biofilms and found that the *srfA* strain is structured but occupies a small footprint, while the double *srfA pgsB* mutant is less structured, similar to the other non-EPS producing strains (Fig. 4d, g). Therefore, as discussed above, the fluid flux into the colony biofilm at early times does not appreciably alter the morphology, and matrix production still governs the structural phenotypes of the mature biofilms.

### PGA production is correlated with high-temperature conditions

**Fig. 5:**
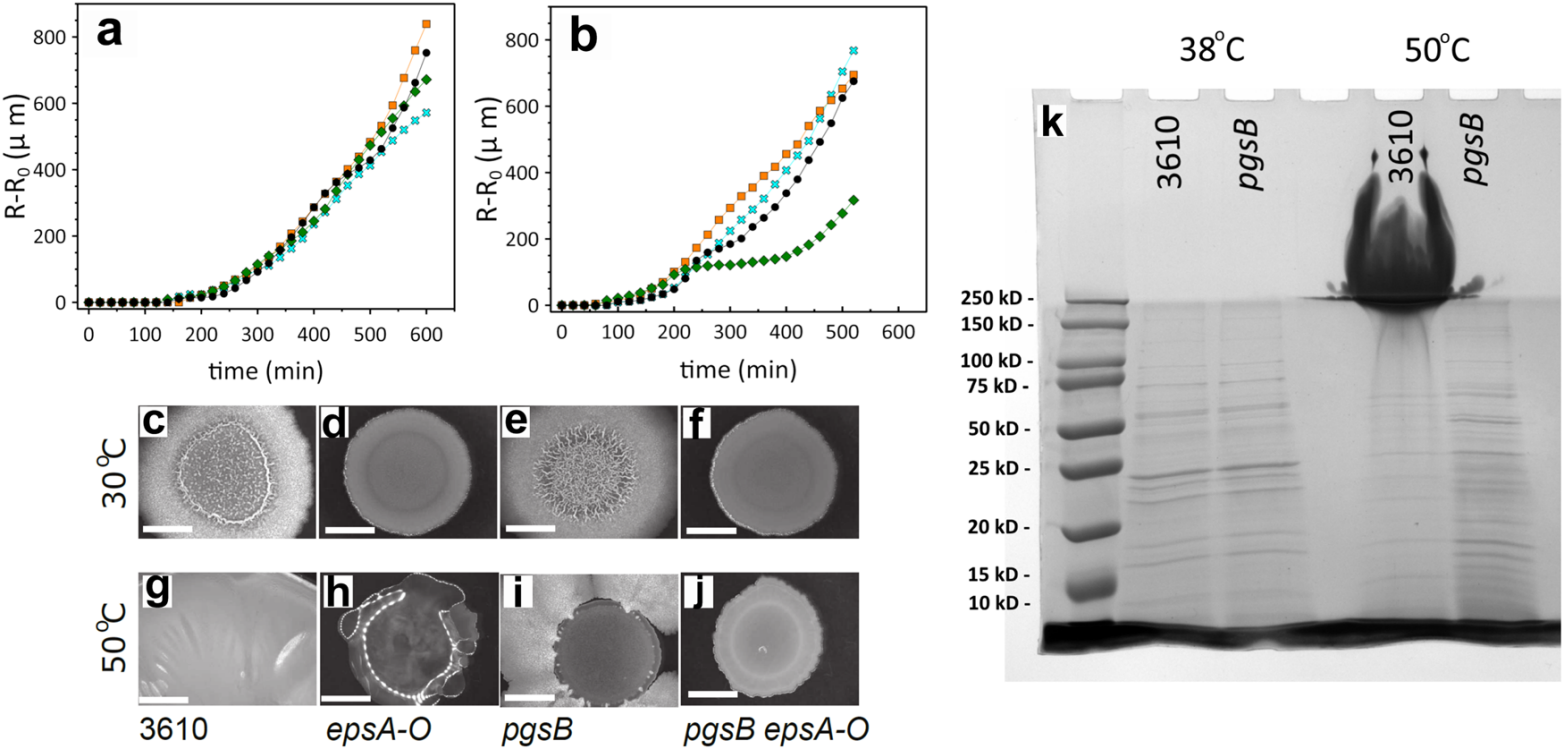
PGA production is temperature dependent. **a** Measurements of the edge expansion at 30 ^∘^C and **b** 42 ^∘^C for NCIB 3610 (black circle), *epsA-O* (green diamond), *pgsB* (orange square), *pgsB epsA-O* (cyan cross). Colony morphology after 48 h incubation at **c**–**f** 30 ^∘^C, and **g**–**j** 50 ^∘^C. Scale bar is 5 mm. **k** Analysis of PGA abundance by polyacrylamide gel electrophoresis of samples extracted from NCIB 3610 and *pgsB* after growth at 38 ^∘^C and 50 ^∘^C.

Our initial analysis was performed at 38 ^∘^C while most other studies investigating *B. subtilis* biofilm formation are typically conducted between room temperature and 30 ^∘^C. We therefore hypothesised that PGA production is not just cell density-dependent, but also temperature-dependent. To test this hypothesis, we repeated the experiments examining fluid flux and colony biofilm morphology with wild-type, *epsA-O*, *pgsB*, and *pgsB epsA-O* strains at the additional temperatures of 30 ^∘^C and 42 ^∘^C (the highest temperature achievable in our microscopy incubator). At 30 ^∘^C we did not observe any fluid flow into the colony biofilm and no fluidisation plateau in the edge expansion for any of the strains, even the wild-type and *epsA-O* strains (Fig. 5a). This result suggests that PGA is not produced at 30 ^∘^C. At the higher temperatures of 38 ^∘^C and 42 ^∘^C, we do find a plateau in the edge expansion curves for the strains able to produce PGA, albeit the plateau at the higher temperature is less pronounced. No plateau is observed for the PGA-deficient mutants (Figs. 4a and 5b).

We additionally found that NCIB 3610 forms exceedingly mucoid colonies on MSgg agar plates at 50 ^∘^C (Fig. 5g). The mucosity was such that when the petri dish was inverted, the biomass dropped onto the lid. We observed similar phenotypes for colonies unable to produce EPS and surfactin (Fig. 5h, Supplementary Fig. 10). We found the mucoid phenotype was directly linked with PGA production as the *pgsB* deletion strains were entirely non-mucoid (Fig. 5i, j). For completion, the same strains were examined at 30 ^∘^C, and no difference in the structure of the NCIB 3610 and *pgsB* colony biofilm architecture was observed (Fig. 5c, e); likewise the *epsA-O* and *pgsB epsA-O* mutants are morphologically comparable (Fig. 5d, f). To support the phenotypic data, we collected the biomass from NCIB 3610 and *pgsB* strains grown at 38 ^∘^C and 50 ^∘^C and biochemically assessed production of PGA. We conclude that 3610 produces high quantities of PGA at 50 ^∘^C but not at 38 ^∘^C (Fig. 5K). Matrix mutants of NCIB 3610 containing deletions in *bslA*, *tasA*, and *epsA-O* form similarly mucoid biofilms at 50 ^∘^C (Supplementary Fig. 10). Only the mutant possessing a deletion in sinR, while still mucoid, produces a biofilm with rugose structural features (Supplementary Fig. 10). Collectively our data support the conclusion that PGA is produced in NCIB 3610 in both a cell density- and temperature-dependent manner, and shows that biofilm architecture and structure can, under certain conditions, be dramatically influenced by the production of PGA.

## Discussion

We have shown that *B. subtilis* produces PGA that induces a fluid flux into a growing biofilm colony. This up-welling of fluid leads to cell flagellar-dependent activity that, in these high density and confined conditions, results in turbulent dynamics. It has been well-established that motile and biofilm matrix-producing cell states are mutually exclusive^41,42^; individual cells can be in only one of the two states. It has also been demonstrated for both Gram-positive and Gram-negative species that active flagellated motility is often required for biofilm development and the role it plays is multi-fold^43–48^.

It is not yet clear what function PGA-induced motility may play in *B. subtilis* biofilm development. It is intriguing that that the same transcriptional regulators required for motility^49^ are also necessary for PGA synthesis^37,50,51^. However, previous work has also shown that there is an *inverse* relationship between PGA synthesis and flagellar function^52,53^, suggesting that the motility we observe may simply be a by-product of the cells being in a fluid environment. The precise regulatory mechanisms that control PGA synthesis and motility are complex and further work is required to better understand the links between the two.

From our experiments it is clear that high temperatures induce the colony to withdraw a considerable volume of fluid from the agar substrate. Previous work has shown that PGA can confer protection to bacteria and increase survival under many different environmental stresses^54–57^. It is plausible that when a biofilm is subject to elevated temperatures, production of PGA would both scavenge and retain moisture which otherwise may be lost through evaporation, thus preventing desiccation of the colony. Our observation of the onset of motility highlights a further potential advantage of PGA production in a changing environment: active (or indeed passive) spreading may facilitate escape and the search for more suitable environs to colonise.

The biofilm matrix of *B. subtilis* and *V. cholerae* has been modelled as a viscous hydrogel network that facilitates biofilm expansion via osmotic fluid influx^58–61^. The localization of EPS production at the propagating boundary of a growing colony is thought to drive the outward expansion of the biofilm. Concomitant production of osmolytes stimulates fluid extraction that swells the matrix at the growing boundary, driving motion forward. In our experiments, PGA seems to have the opposite effect: colony expansion stalls due to the colony entering a ‘liquid-like’ state when fluid is extracted from the substrate. Previous work has shown that colony biofilm expansion is strongly governed by mechanical contact forces between neighbouring cells and friction with the underlying substrate^62–64^. In our experiments, expansion only recommences when the fluid environment dissipates, physical contacts are restored, and non-PGA matrix production begins. This raises a further question: how does colony expansion occur when PGA is the primary matrix component? Our experiments at high temperatures show that the wild-type 3610 strain does expand significantly beyond the initial deposition footprint. Strains that do not produce EPS or surfactin do not expand as much as the wild-type hinting that these components have a role to play in facilitating colony expansion (Supplementary Fig. 10).

The travelling waves of fluid in the *epsA-O* deficient strain results in the appearance of finger-like structures as the wave propagates inwards. Such fingering instabilities can occur when a low viscosity fluid displaces one of a higher viscosity; the inverse situation will typically result in a stable interface. Curiously, in our experiments the fingers occur in the inverse configuration. Such inverse Saffman-Taylor instabilities can occur by the addition of wettable particles that can adsorb to the air/fluid interface and induce interfacial instabilities^65^. It is known that bacteria can accumulate at interfaces^66^, and *B. subtilis* demonstrates this by forming floating (pellicle) biofilms. We speculate that in our experimental system, the *B. subtilis* cells accumulate at the front of the incoming fluid wave, modifying the interfacial energetics and destabilising the interface between the fluid and the air, and giving rise to the observed fingering instability. Localisation of cells to these interfaces may also give rise to cell density gradients, which in turn may evolve as patterns in the mature biofilm. More work will need to be done to uncover the biological and physical mechanisms that cause this unusual phenomena and any possible benefit or function in ecological settings.

Our experiments at intermediate temperatures (38 ^∘^C) are suggestive of a spatial separation between PGA-producing cells at the edge of the colony biofilm and matrix-producing cells in the middle (as has previously been reported^67^), resulting in the annular confinement of fluid. Such spatial and temporal heterogeneity is a common feature in biofilms where the local microenvironment can strongly influence the phenotypic state of the cells. Phenotypic heterogeneity within a biofilm can be generated from variations in the chemical or physical environment^68,69^, genotypic variations, and stochastic gene expression^68^.

The heterogeneity in cell density imposed by the initial deposition conditions—and the formation of the ‘coffee ring’—leads to the spatial pattern of fluid extraction that we observe. However, this is not the only means of generating density differences within a biofilm. Aggregates formed while growing in liquid culture can seed patches of higher cell density across the deposition footprint. Indeed, we observe that fluid invasion and, by inference, PGA production can occur in small localised regions far away from the ‘coffee ring’ (Supplementary Movie 23). This suggests that there is some critical cell density that determines whether an individual cell adopts a PGA-producing state over a matrix-producing one.

**Fig. 6:**
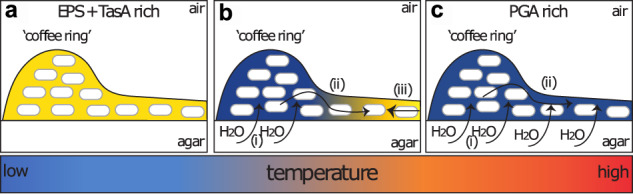
Schematic model of matrix production as a function of density and temperature. The `coffee ring' is the initial region of higher density that is formed after deposition onto the agar surface. **a** Low temperatures produce a biofilm matrix rich in EPS and TasA (yellow). **b** Intermediate temperatures induces PGA production (blue) and (i) concomitant fluid extraction from the agar that originates in the high density region. (ii) The fluid propagates towards the center where (iii) EPS and TasA matrix production halts its advance. **c** High temperatures results in a PGA rich matrix that induces (i) fluid extraction that (ii) covers the entire biofilm resulting in a mucoid phenotype.

The spatial heterogeneity is transient at 38 ^∘^C, and EPS becomes the dominant matrix component that determines the large-scale biofilm morphology. The fluid flux is ultimately stopped by the production of the EPS element of the matrix in the middle region of the biofilm (Fig. 6b). The motile cells that are close to the EPS-producing cells appear to abruptly stop moving, and a solid front rapidly advances from the middle of the colony outwards. One mechanism that could explain this transition involves engagement of a “molecular clutch" like EpsE, which binds to FliG and triggers motility arrest^70^. However, we can rule out EpsE as a candidate since we observe propagation of the motility arrest front in the *epsA-O* strain. It remains an open question of what physical or biological mechanism(s) govern this switch-like phenomenon.

At low temperatures we did not observe the phenotypic heterogeneity that we observe at intermediate temperatures, presumably due to a lack of PGA production (Fig. 6a) despite the high cell density in the coffee ring. Conversely, at high temperatures biofilms are extraordinarily mucoid and lack any discernible structure typical of a *B. subtilis* biofilm (Fig. 6c). In either extreme, any cell density-dependent PGA production is superseded by a temperature-dependent pathway which impacts the entire biofilm.

When engineered to overproduce the TasA fibres and exopolysaccharide by the introduction of a mutation in *sinR*, the biofilms formed at high temperature (Supplementary Fig. 10), were highly mucoid but also possessed a morphology more typical of a wild-type *B. subtilis* biofilm. This implies that the production of PGA can occur simultaneously with EPS/TasA, even if individual cells in the population commit to the production of one or other product. This bifurcation into two populations is suggested by our findings at intermediate temperatures, where the two cell types appear to be present at the same time, but in different regions of the biofilm.

Previous work has shown that strains possessing a deletion of *spo0A* result in biofilms that are mucoid and unstructured at low temperatures (30 ^∘^C) and that this was due to PGA production^27,71^. Therefore, a *spo0A* mutant broadly mimics the wild-type biofilm phenotype that we observe at high temperatures. This implies that Spo0A may be the regulatory component that controls temperature-dependent production of a PGA-rich or EPS/TasA-rich biofilm matrix.

Taken together, these results imply that *B. subtilis* has the ability to produce different biofilm matrices with distinct properties to adapt to disparate environmental conditions. Such a strategy may be employed in natural environments given *B. subtilis* can grow at a wide range of temperatures: it is found in desert soils and within composts which can easily reach temperatures of 50 ^∘^C. Until now, PGA was not thought to be an important factor as a matrix component in *B. subtilis* NCIB 3610 biofilms^37^. However, it appears, under the right conditions, PGA can become an alternative matrix component with distinct structural and physical characteristics that may aid the biofilm to survive in high temperature conditions.

## Methods

### Growth conditions

*B. subtilis* strains were initially grown in LB medium (10 g NaCl, 5 g yeast extract, and 10 g tryptone per liter). Biofilms were grown on MSgg agar (5 mM potassium phosphate and 100 mM MOPs at pH 7.0 supplemented with 2 mM MgCl_2_, 700 μM CaCl_2_, 50 μM MnCl_2_, 50 μM FeCl_3_, 1 μM ZnCl_2_, 2 μM thiamine, 0.5% v/v glycerol, 0.5% w/v glutamate, 1.5% w/v Select Agar (Invitrogen). When appropriate, antibiotics were used as required at the following concentrations: chloramphenicol at 5 μg ml^−1^, kanamycin at 25 μg ml^−1^, spectinomycin at 100 μg ml^−1^, and tetracycline at 10 μg ml^−1^.

### Strain construction

All strains used in this study are provided in Supplementary Table 1. All *B. subtilis* strains used in this work are derived from the wild-type laboratory isolate NCIB 3610 and constructed using standard protocols. SPP1 phage transduction was used for transfer of genomic DNA from the donor strain into the recipient NCIB 3610.

### Extraction and detection of poly-*γ*-glutamic acid

Colony biofilms were grown for 24 h, at which point the biomass was harvested from the agar surface and placed into a 500 μl aliquot of BugBuster^*Ⓡ*^ Mastermix (Merck). The material was disrupted via repeated passage through a 23-gauge needle and then sonication (30% amplitude, 10 s). The samples were incubated at 21 ^∘^C (with agitation) for 20 min and centrifuged at 4 ^∘^C (17,000 × *g*) for 10 min. The supernatant was retained and contained PGA and extracted proteins. The samples were quantified using a Pierce BCA protein assay kit (Thermo Scientific) and stored at −20 ^∘^C prior to further analysis.

Samples containing 10 μg of total protein (normalised to 25 μl in 1x laemelli sample buffer) were analysed by SDS-PAGE (12% w/v resolving gel and 7% w/v stacking gel) alongside a molecular weight ladder (Precision Plus Protein Dual Colour Standards (Biorad)). All gels were processed in parallel and were run at 180 V until the blue dye front in the sample loading dye reached the bottom of the gel. The acrylamide gels were thoroughly rinsed in water and soaked in Instant Blue Coomassie protein stain (Abcam) for 1 h with gentle rocking. After incubation in the stain, the gels were rinsed with deionized water and imaged using a Molecular Imager Gel Doc XR system (Biorad). The gels were further soaked in 0.5% (w/v) methylene blue in 3% (v/v) acetic acid for 10 min then repeatedly washed in deionized water until background non-bound dye was removed.

### Biofilm imaging and analysis

*B. subtilis* strains were inoculated into 3 mL LB from a single colony grown on 1.5% w/v LB agar. The bacteria were allowed to grow at 30 ^∘^C with 200 rpm orbital shaking until reaching an OD between 1.5 and 2. The cell culture was diluted to OD_600_ 1.0 in phosphate-buffered saline. A 3 μL droplet of bacteria was deposited onto a 35 mm petri dish (Corning) containing MSgg agar. The droplets of bacteria were allowed to dry for 10 min. For samples prepared via spin-coating, a petri dish containing MSgg agar was placed onto a vacuum spin-coater (Cammax Precima) rotating at 2000 rpm. A 10 μL droplet of bacteria was placed on the rotating agar. For all experiments, the petri dish was placed on a Nikon Ti inverted microscope that is temperature controlled. All microscopy images and movies were captured using a CoolSNAP HQ2 CCD camera controlled by *μ*Manager software. Brightfield movies and images of the biofilms were captured from the underside of the petri dish and are imaged through the agar (thickness ~ 4 mm). Nikon Plan 2x UW and 10X Plan Fluor objectives were used in image acquisition. Additional biofilm imaging was performed using a Leica MZ16 stereoscope. For the bead tracking experiments, 1 μm diameter latex carboxylate-modified polystyrene yellow fluorescent beads (Sigma-Aldrich) were diluted into PBS from the stock solution in a 1:1000 ratio. 1 μL of the working solution was added to the 1 mL of the diluted cell culture just prior to deposition. Movies were acquired in bright and epifluorescent channels (Nikon GFP fluorescent filter cube) and the beads were tracked using the ImageJ plugin TrackMate (v3.8.0)^72^. Image analysis was performed using the Fiji distribution of ImageJ. Whole biofilm microscopy images were captured as above but in multiple tiles that were stitched together using the ‘Pairwise Stitching’ plug-in. The images of the fingering instabilities were generated by first using the ‘straighten’ tool to transform a circular to a linear region. The default ImageJ threshold method was applied to binarize the images. In cases where the intensity varied across the image, the image was partitioned into regions of similar intensity and then thresholding was performed. The ’edge finding’ tool was used to locate the edges of the thresholded images. After edges were identified the interior was filled to form a representation of the fingers. If the image was partitioned, the image was stitched together using the stitching tool in ImageJ. Measurement of fluid travel distance was manually tracked in ImageJ and the mean displacement was averaged over 10 separate measurements for each experiment. For each strain studied, three separate experiments were performed. Edge and front displacement measurements were similarly performed in ImageJ by manually tracking the movement of the front over successive frames. Displacements were always measured relative to the initial position of each feature. PIV analysis was performed using PIVlab (v2.39)^73,74^. Images were first processed using a CLAHE filter with a window size of 20 pixels. Three interrogation passes were used with window sizes of 64/32 pixels, 32/16 pixels, and 16/8 pixels. The subpixel estimator used was the Gauss 2x3-point. Image masks were applied to limit spurious vector estimation and vectors were rejected if they exceeded 5 standard deviations from the mean.

### Reporting summary

Further information on research design is available in the Nature Research Reporting Summary linked to this article.

## Supplementary information

Supplementary Information

Reporting Summary

Supplementary Movie 1

Supplementary Movie 2

Supplementary Movie 3

Supplementary Movie 4

Supplementary Movie 5

Supplementary Movie 6

Supplementary Movie 7

Supplementary Movie 8

Supplementary Movie 9

Supplementary Movie 10

Supplementary Movie 11

Supplementary Movie 12

Supplementary Movie 13

Supplementary Movie 14

Supplementary Movie 15

Supplementary Movie 16

Supplementary Movie 17

Supplementary Movie 18

Supplementary Movie 19

Supplementary Movie 20

Supplementary Movie 21

Supplementary Movie 22

Supplementary Movie 23

## Data Availability

All data used in this study will be deposited in the Edinburgh DataShare (10.7488/ds/3473). Any data are also available upon request to the corresponding authors.
