# Supplementary material for: Density and temperature controlled fluid extraction in a bacterial biofilm is determined by poly-γ-glutamic acid production
Source: NPJ Biofilms Microbiomes. 2022 Dec 17;8:98. doi: 10.1038/s41522-022-00361-5 (PMC9759580; doi:10.1038/s41522-022-00361-5)
Supplement: Supplementary file 1 — Supplementary Information [file 41522_2022_361_MOESM1_ESM.pdf]

Supplementary Information: Density and temperature controlled fluid extraction in a bacterial biofilm is determined by poly-γ-glutamic acid production

Ryan J. Morris[a], David Stevenson[b], Tetyana Sukhodub[b], Nicola R. Stanley-Wall[b] and Cait E. MacPhee[a]

[a]National Biofilms Innovation Centre, School of Physics and Astronomy, The University of Edinburgh, Edinburgh EH9 3FD, United Kingdom; [b]Division of Molecular Microbiology, School of Life Sciences, University of Dundee, Dundee DD1 5EH, United Kingdom

Corresponding Authors Email: ryan.morris@ed.ac.uk; n.r.stanleywall@dundee.ac.uk; cait.macphee@ed.ac.uk

Supplementary Figures

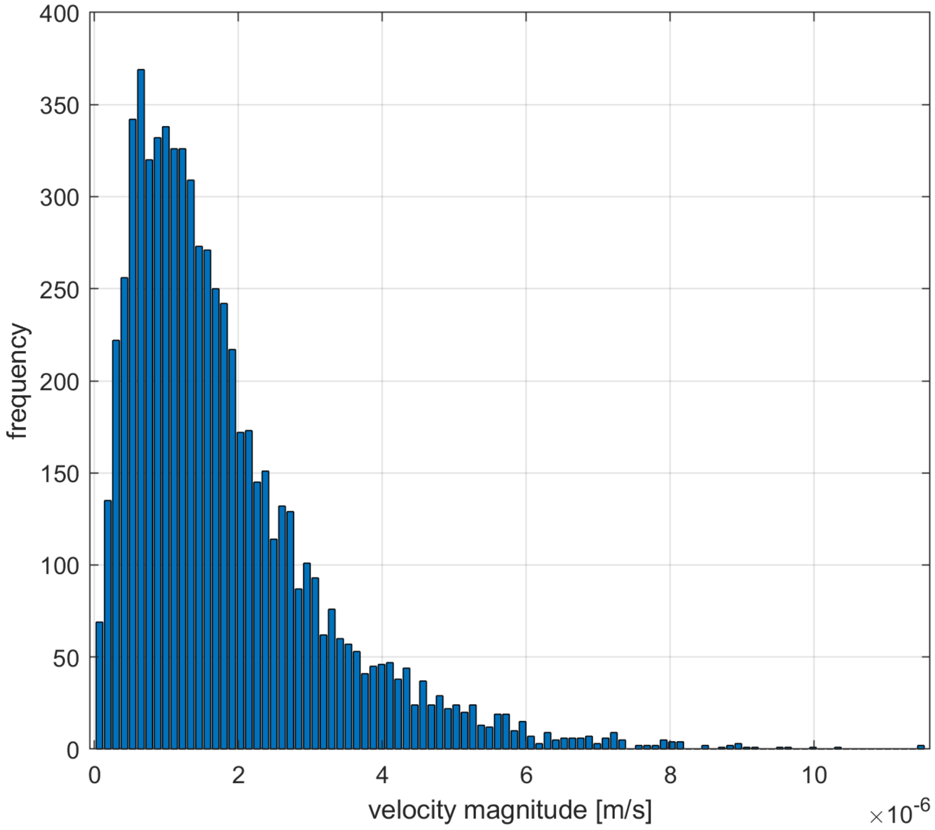

Supplementary Figure 1. Representative velocity magnitude histogram for strain NCIB 3610 obtained from PIV analysis. Data acquired $\sim$ 4.5 hours after deposition (Supplementary Movie 3).

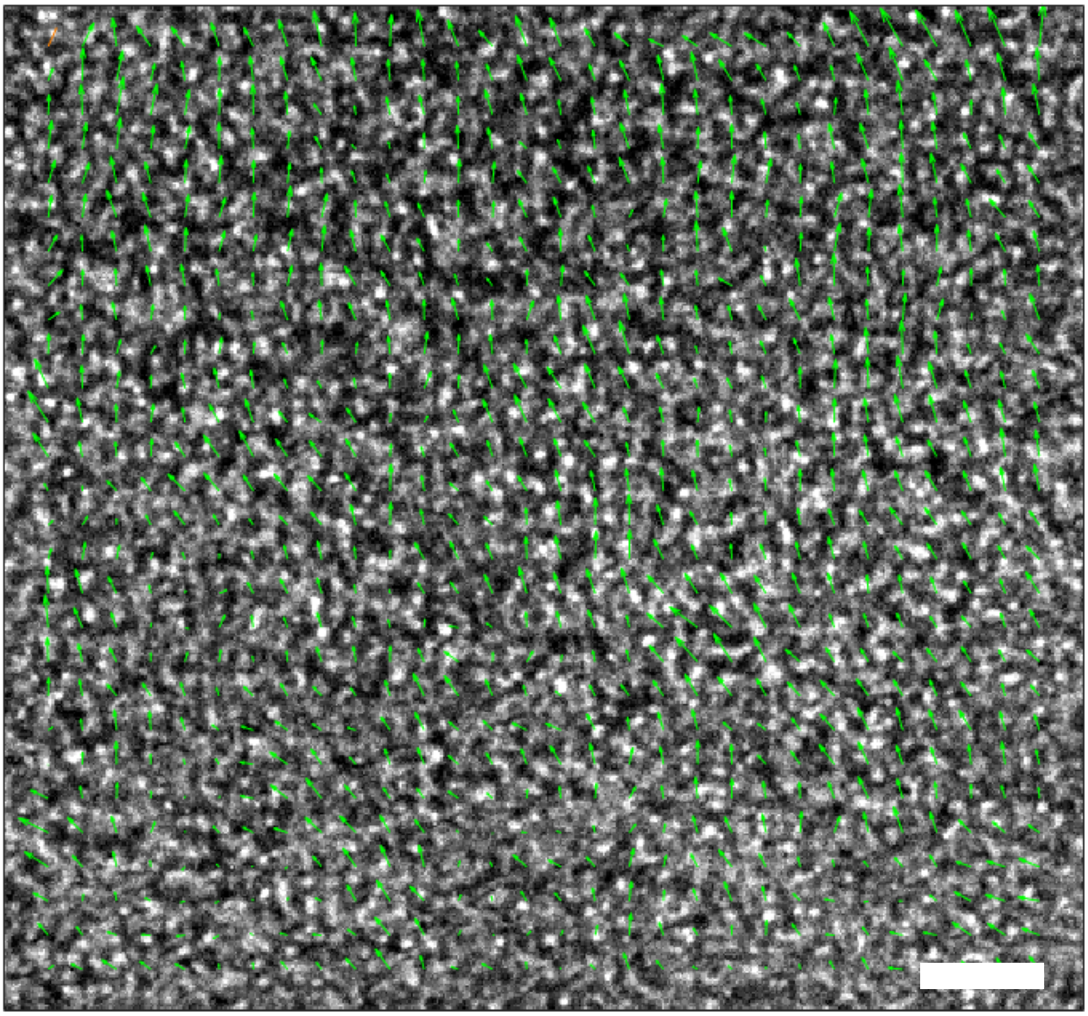

Supplementary Figure 2. Representative velocity map for *motB* strain obtained from PIV analysis. Scale bar is 100μm.

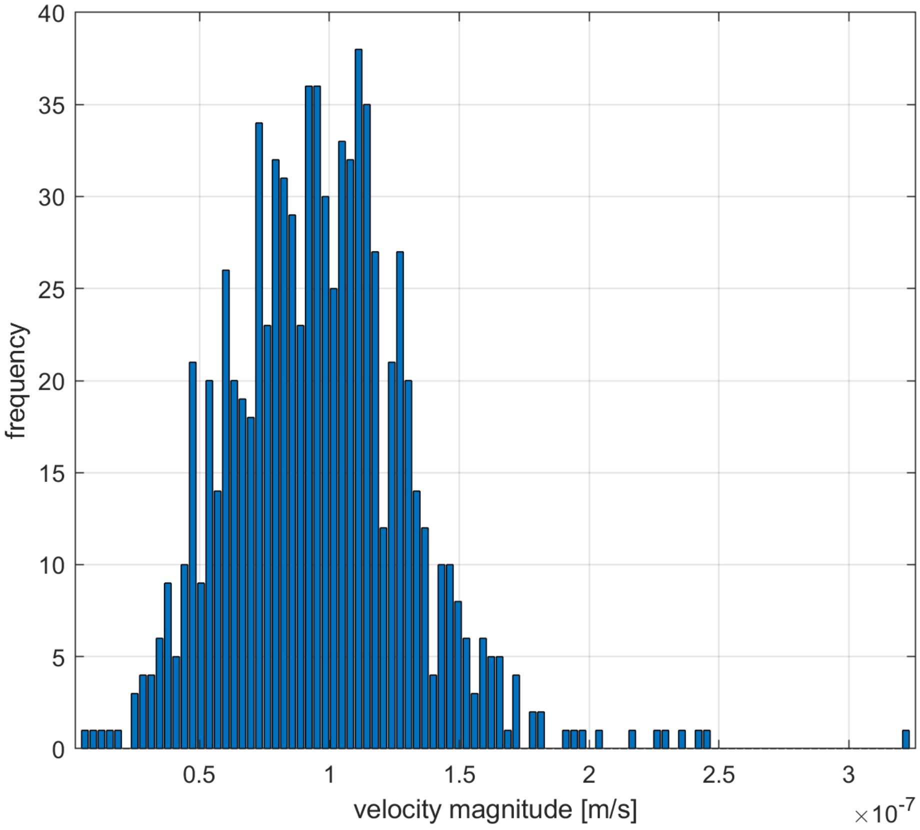

Supplementary Figure 3. Representative velocity magnitude histogram for *motB* strain obtained from PIV analysis.

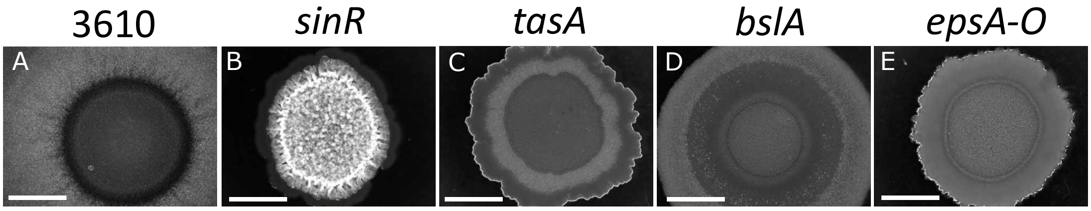

Supplementary Figure 4. Representative colony biofilm morphology of matrix deficient strains at 38°C after 48 hours incubation. Scale bars are 5 mm.

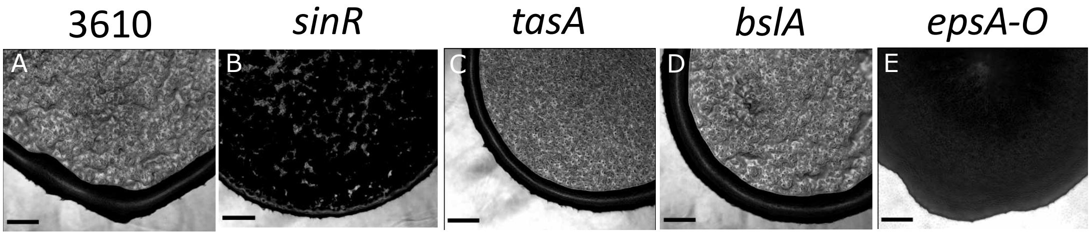

Supplementary Figure 5. Microscopy images of matrix deficient strains taken from below through the agar at 38°C. Scale bar is 200 μm

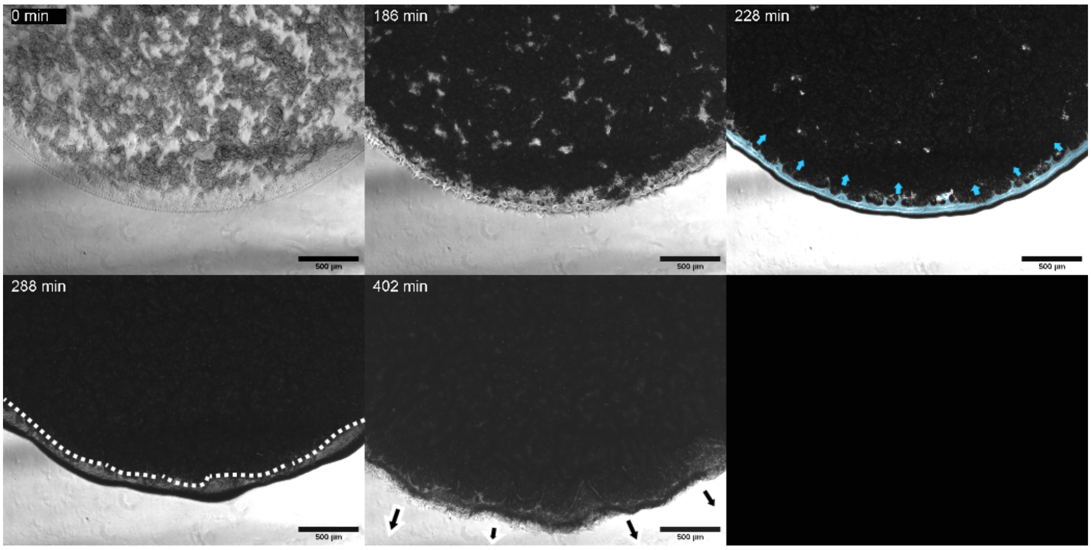

Supplementary Figure 6. Still images taken from Movie S9 of the *sinR* strain. At t=0 min shows the bacteria just after deposition. T=186 min the bacteria have grown and there is a noticeable higher density of cells at the periphery of the colony. T=228 min we observe influx of fluid highlighted in blue; blue arrows indicate movement of fluid which is highly restricted for *sinR*. T=288 min, the maximum extent of fluid has pushed into the interior (white dotted line). T=402 min, the colony is now expanding outwards.

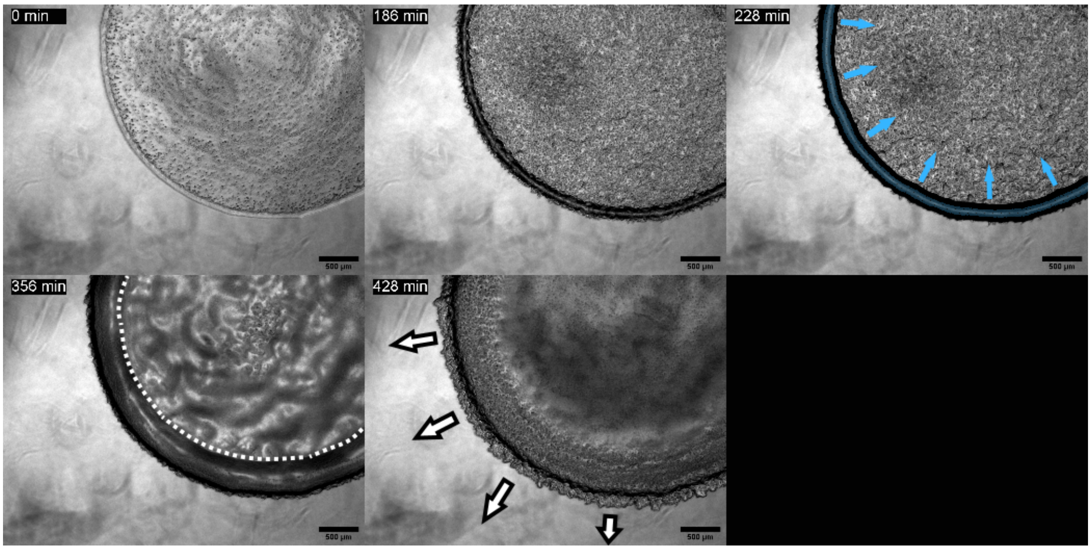

Supplementary Figure 7. Still images taken from Movie S10 of *bslA* strain. At t=0 min shows the bacteria just after deposition. T=186 min the bacteria have grown and there is a noticeable higher density of cells at the periphery of the colony. T=228 min we observe influx of fluid highlighted in blue; blue arrows indicate movement of fluid. T=288 min, the maximum extent of fluid has pushed into the interior (white dotted line). T=428 min, the colony is now expanding outwards.

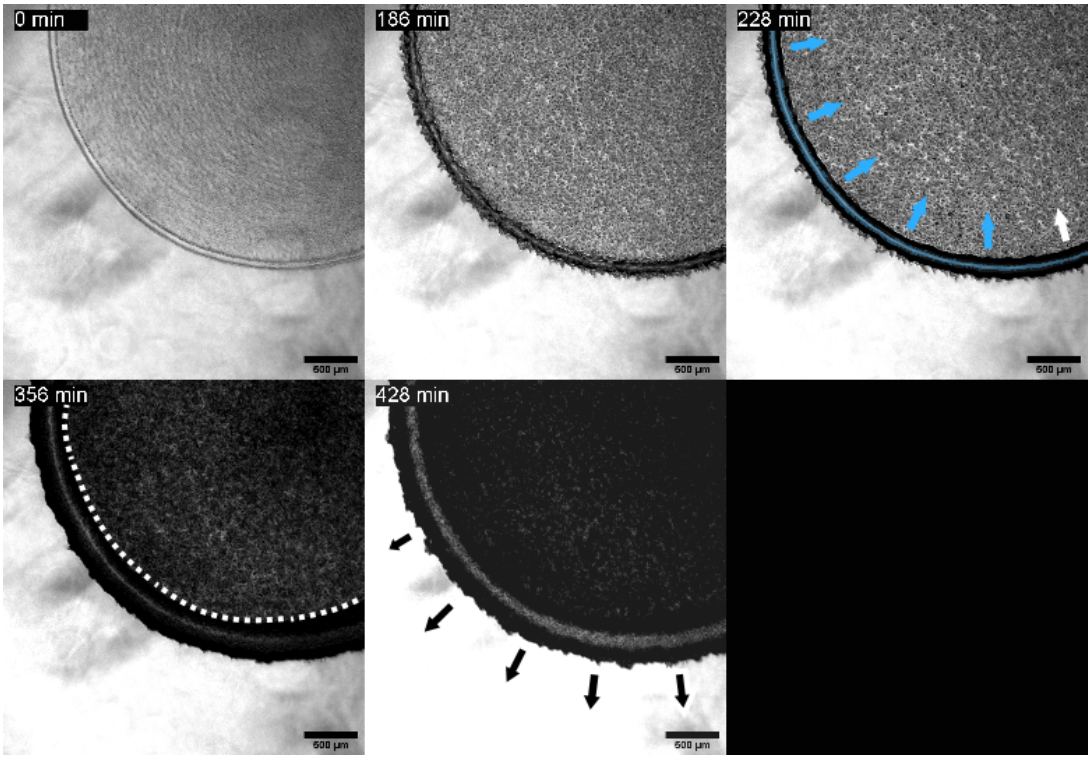

Supplementary Figure 8. Still images taken from Movie S11 of *tasA* strain. At t=0 min shows the bacteria just after deposition. T=186 min the bacteria have grown and there is a noticeable higher density of cells at the periphery of the colony. T=228 min we observe influx of fluid highlighted in blue; blue arrows indicate movement of fluid. T=288 min, the maximum extent of fluid has pushed into the interior (white dotted line). T=428 min, the colony is now expanding outwards.

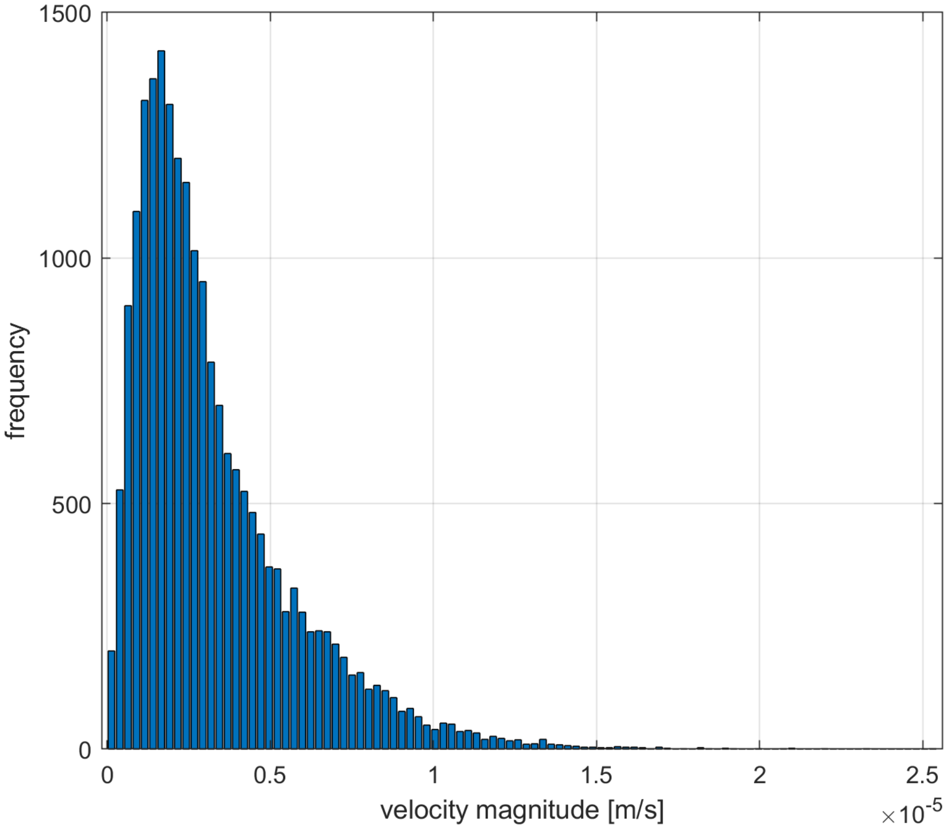

Supplementary Figure 9. Representative velocity magnitude histogram for *epsA-O* strain obtained from PIV analysis. Data acquired $\sim$ 5 hours after deposition (see Supplementary Movie 11).

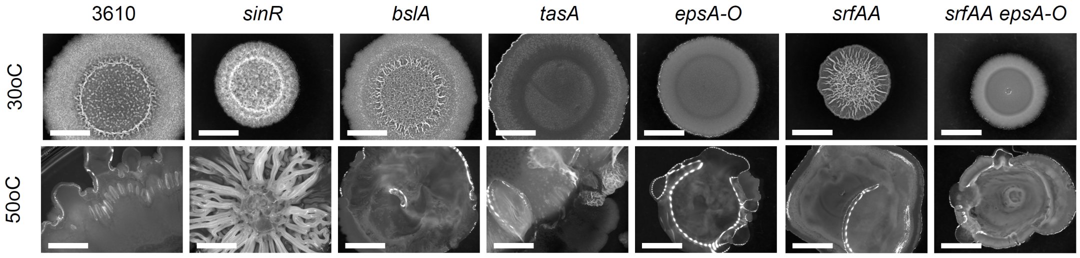

Supplementary Figure 10. Representative colony biofilm morphology of matrix and / or surfactin deficient strains at 30°C and 50°C after 48 hours incubation. Scale bars are 5 mm.

Supplementary Tables

Strain	Genotype	Reference / Construction
NCIB 3610	wild-type prototroph	BGSC
NRS2415	Δ*tasA*::spc	[1]
NRS2450	Δ*epsA-O*::tet	[2]
NRS2097	Δ*bslA*::cml	[1]
NRS1859	Δ*sinR*::kan	[3]
NRS6962	Δ*srfAA*::kan Δ*epsA-O*::tet	NRS2450 → NRS6962
NRS6958	Δ*srfAA*::kan	BKK03480 → NCIB 3610
NRS7014	Δ*pgsB*::spc	BAL1811 → NCIB 3610
NRS7015	Δ*pgsB*::spc Δ*epsA-O*::tet	BAL1811 → NRS2450
NRS3494	3610 Δ*motB*	pNW654 → NCIB 3610[4]
BAL1811	JH642 *trpC2 pheA1* Δ*pgsB*::spc	[5]
BKK03480	Δ*srfAA*::kan	[6]

Supplementary Table 1. Table of strains used in this work. Drug resistance cassettes are indicated as follows: cml, chloramphenicol resistance; kan, kanamycin resistance; tet, tetracycline resistance; and spc, spectinomycin resistance. BGSC represents the Bacillus genetic stock center. The direction of strain construction is indicated with phage SPP1 (→) recipient strain.

Supplementary References

[1] Adam Ostrowski, Angela Mehert, Alan Prescott, Taryn B Kiley, and Nicola R Stanley-Wall. Yuab functions synergistically with the exopolysaccharide and TasA amyloid fibers to allow biofilm formation by *Bacillus subtilis*. *Journal of bacteriology*, 193(18):4821–4831, 2011.

[2] Steven S Branda, Frances Chu, Daniel B Kearns, Richard Losick, and Roberto Kolter. A major protein component of the *Bacillus subtilis* biofilm matrix. *Molecular microbiology*, 59(4):1229–1238, 2006.

[3] Taryn B Kiley and Nicola R Stanley-Wall. Post-translational control of *Bacillus subtilis* biofilm formation mediated by tyrosine phosphorylation. *Molecular microbiology*, 78(4):947–963, 2010.

[4] Lynne S Cairns, Victoria L Marlow, Emma Bissett, Adam Ostrowski, and Nicola R Stanley-Wall. A mechanical signal transmitted by the flagellum controls signalling in *Bacillus subtilis*. *Molecular Microbiology*, 90(1):6–21, 2013.

[5] Nicola R Stanley and Beth A Lazazzera. Defining the genetic differences between wild and domestic strains of *Bacillus subtilis* that affect poly-γ-dl-glutamic acid production and biofilm formation. *Molecular microbiology*, 57(4):1143–1158, 2005.

[6] Byoung-Mo Koo, George Kritikos, Jeremiah D Farelli, Horia Todor, Kenneth Tong, Harvey Kimsey, Ilan Wapinski, Marco Galardini, Angelo Cabal, Jason M Peters, et al. Construction and analysis of two genome-scale deletion libraries for *Bacillus subtilis*. *Cell systems*, 4(3):291–305, 2017.
